# Analysis of Laparoscopic Ultrathin Choledochoscope Curative Effect on Common Bile Duct Exploration and Choledocholithotomy in 47 Cases

**DOI:** 10.3389/fsurg.2022.782357

**Published:** 2022-03-31

**Authors:** Yang Liu, Tao Yang, Jia-Hong Liu, Xuan Meng, Hong-Tian Xia

**Affiliations:** ^1^Faculty of Hepato-Pancreato-Biliary Surgery, The First Medical Center, Chinese People's Liberation Army General Hospital, Beijing, China; ^2^Department of Hepatobiliary Surgery, Weifang Traditional Chinese Medicine Hospital, Weifang, China

**Keywords:** choledocholithiasis, laparoscopic cholecystectomy, Laparoscopic Transcystic Common Bile Duct Exploration, ultrathin choledochoscope, bile duct exploration

## Abstract

**Objective:**

The aim of the present study is to summarize the experience of using a 2. 7 mm choledochoscope for laparoscopic cholecystectomy combined with an ultrathin choledochoscope for common bile duct exploration and choledocholithotomy in the treatment of cholecystolithiasis associated with choledocholithiasis after the implementation of strict inclusion and exclusion criteria.

**Methods:**

A retrospective analysis of 47 patients with cholecystolithiasis complicated with choledocholithiasis who were treated in the hepatopancreatobiliary surgery department of the Chinese People's Liberated Army General Hospital between January 2015 and December 2019 was performed in the present study. Clinical data of laparoscopic cholecystectomy combined with ultrathin choledochoscope transcystic duct exploration for common bile duct and choledocholithotomy.

**Results:**

All 47 patients completed the operation successfully. The gallbladder duct was closed using a surgical clamp. Only 2 patients were administered with an abdominal drainage tube. The operation time was 50–160 min, the intraoperative blood loss was 5–50 ml, and the postoperative hospital stay was 2–8 days. No patients had serious complications, such as bile leakage, postoperative bleeding, cholangitis, biliary pancreatitis, and wound infection. Minor complications, such as abdominal pain (Abdominal pain was defined as a patient felt tolerable or unbearable abdominal pain but improved or disappeared with medication) and diarrhea, were present in a few patients; these improved after conservative treatment. There was no recurrence of calculi during the 1–5 years of follow-up, and the patient quality of life was good.

**Conclusion:**

Laparoscopic cholecystectomy combined with ultrathin choledochoscope common bile duct exploration and choledocholithotomy is a safe and effective method after adopting strict inclusion and exclusion criteria. This technology was started in the First Medical Center, Chinese People's Liberation Army General Hospital in September 2009, and it has become extremely mature in the past 5 years.

## Background

At present, the main surgical methods for the treatment of cholecystolithiasis and choledocholithiasis in China are (1) single-stage: Laparoscopic cholecystectomy combined with laparoscopic common bile duct exploration or (2) two-stage: endoscopic retrograde cholangiopancreatography combined with endoscopic sphincterotomy after laparoscopic cholecystectomy. Compared with the two-step method, the one-step method not only requires fewer steps (1), but can also reduce the risk of stone recurrence, cholangitis, and pancreatitis occurrence (2–4).

Therefore, one-step surgical methods are used for the treatment of cholecystolithiasis combined with choledocholithiasis in the First Medical Center, Chinese People's Liberation Army General Hospital. There are two approaches to common bile duct exploration and choledocholithotomy in the First Medical Center, Chinese People's Liberation Army General Hospital: (1) cutting the common bile duct for extraction and (2) using ultrathin choledochoscope (outer diameter = 2.7 mm) for common bile duct exploration and choledocholithotomy. Because ultrathin choledochoscope is unique in its use, patients who undergo ultrathin choledochoscope treatment also need to choose. The inclusion methods are described in Patients and methods.

In recent years, the First Medical Center, Chinese People's Liberation Army General Hospital has reported this technology in two articles (5, 6). An analysis of the safety and efficacy in patients treated with this technology will be conducted after the publication of these two articles.

## Patients and Methods

The present study has been approved by Ethics Committee of the First Medical Center, Chinese People's Liberation Army General Hospital. As this is a retrospective study, informed consent was not required. The diagnosis of cholecystolithiasis was conducted via abdominal ultrasound, magnetic resonance cholangiopancreatography, and computed tomography. The patient clinical history, clinical symptoms, and imaging findings were used to diagnose choledocholithiasis. All patients gave their informed consent to the procedure.

Inclusion criteria: (1) Patients aged 20–80 years; (2) patients with cardiopulmonary function assessed preoperatively to determine laparoscopic surgery tolerance; (3) patients with a confirmed diagnosis of cholecystolithiasis complicated with choledocholithiasis; (4) patients who underwent abdominal Magnetic Resonance Cholangiopancreatography (MRCP) and ultrasound examination before surgery; (5) patients with a choledocholithiasis diameter of ≤ 1 cm and ≤ 1.5 times the diameter of the cystic duct (in the case of a choledocholithiasis diameter of >1 cm, the choledocholithiasis diameter will be >5 mm; a 5 mm choledochoscope can be used, thus it is not discussed in this article); The diameter of the cystic duct was measured by preoperative Magnetic Resonance Cholangiopancreatography (MRCP). If the cystic duct that is too thin or folded cannot be measured by MRCP, a visual judgment is made during the operation: the inclusion criteria can be excluded if it can pass through a 5 mm choledochoscope. And (6) patients with no prior abdominal surgery or abdominal surgery that does not affect laparoscopic cholecystectomy (Abdominal surgery that does not affect laparoscopic cholecystectomy' refers to the process from the establishment of the surgical puncture to the completion of cholecystectomy, and the separation of the adhesions generated by the previous surgery does not affect the final completion of the surgery). The choledocholithiasis number was unlimited.

Exclusion criteria (6): (1) Patients with primary cholangiolithiasis; (2) patients with cholecystolithiasis combined with acute cholecystitis (not chronic cholecystitis); (3) patients with severe obstructive jaundice; (Total bilirubin > 100 μmol/L, liver function Child-Pugh grade B or lower). (4) patients combined with intrahepatic cholangiolithiasis; (5) patients with cystic bile duct dilatation combined with choledocholithiasis; (6) patients with abnormal duodenal papilla function; (7) patients with recurrent choledocholithiasis following endoscopic retrograde cholangiopancreatography and endoscopic sphincterotomy; (8) patients with an anomalous pancreaticobiliary duct arrangement; and (9) patients with contraindications of intolerant pneumoperitoneum and other laparoscopic surgery.

General laparoscopic equipment and instruments are used in the procedure. The choledochoscope uses the Olympus CB30 with an outer diameter of 2.7 mm, which is an ultrathin choledochoscope equipped with a corresponding stone collection basket. The holmium laser lithotripsy combined with an ultrathin choledochoscope was used for surgery from 2009 to 2014. Since 2015, ultrathin choledochoscope technology can only be used after the adoption of strict inclusion and exclusion criteria. Thus, the use of holmium laser lithotripsy was no longer included in the present case summary.

**Figure 1:**
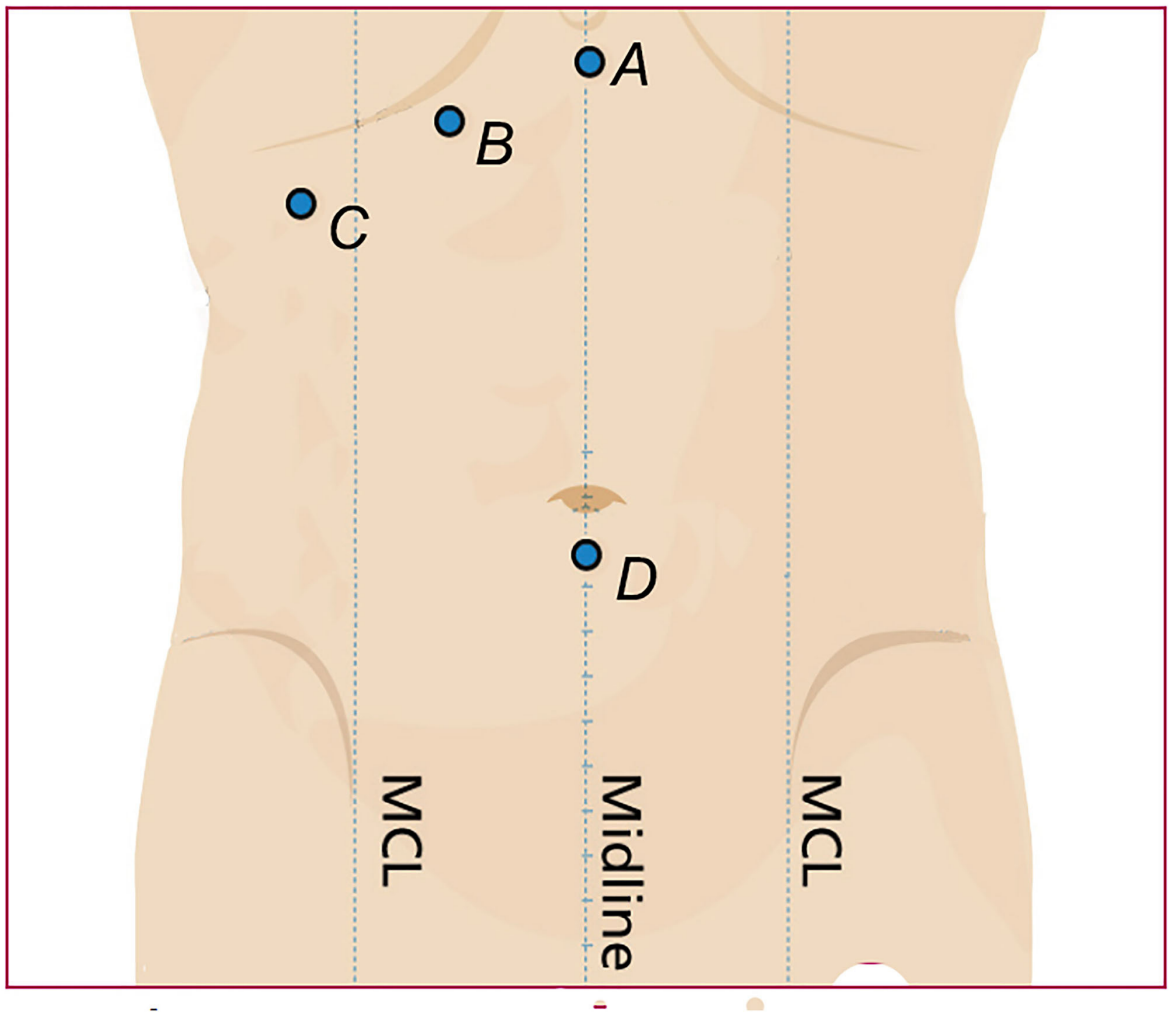
**(A)** Subxiphoid Trocar, **(B)** The working passage of the choledochoscope along the subcostal margin of the right clavicular line. **(C)** An auxiliary hole placed on the axillary front along the lower margin of the rib. **(D)** The laparoscopic camera port below the umbilical.

All patients received tracheal intubation and general anesthesia, and the surgery was performed by an experienced surgical team. The “four-hole method” is used routinely. Three 10–12 mm trocars were inserted below the xiphoid process along the subcostal margin of the right clavicular line (the working passage for the choledochoscope) and below the umbilical region (the laparoscopic camera port). A 5 mm trocar was placed on the axillary front along the lower margin of the rib as an auxiliary hole (6) (Figure 1).

**Figure 2:**
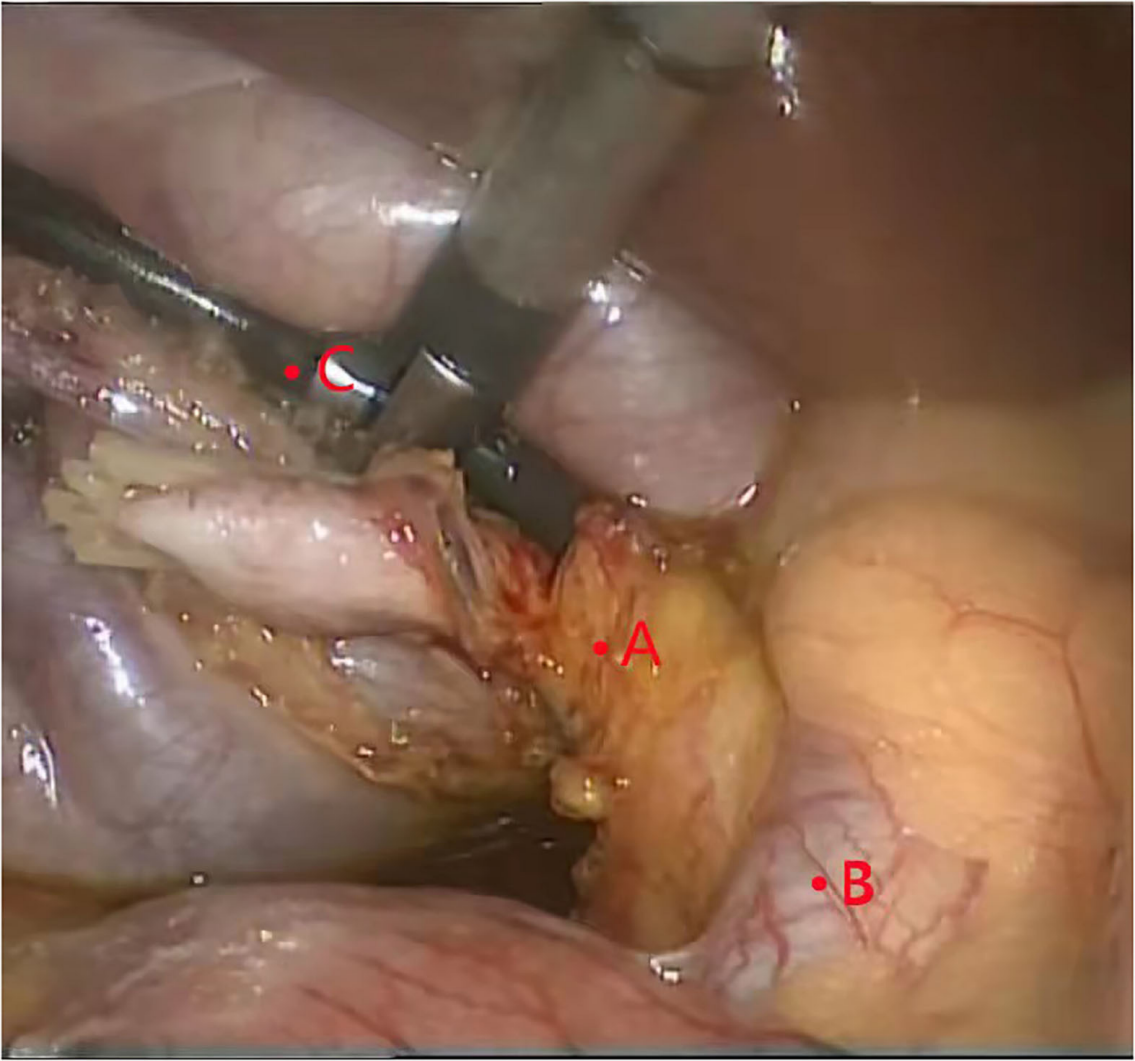
Using ultrathin choledochoscope to remove broken stones. **(A)** cystic duct. **(B)** common bile duct. **(C)** ultrathin choledochoscope.

First, the gallbladder triangle was dissected, and the cystic artery and bile duct were dissected and exposed. The gallbladder artery was then ligated and severed, and the cystic duct was completely dissociated to the confluence of the cystic duct and common hepatic duct. A surgical clamp was used to close the cystic duct near the ampulla of the gallbladder to prevent bile and stones from entering the duct during the operation. A transverse incision with a length of 1/3–1/2 of the cystic duct enlargement was made at 3–5 mm from the common bile duct. A 2.7 mm ultrathin choledochoscope was selected to enter the common bile duct through the cystic duct for exploration, and the stone was removed (Figure 2).

**Figure 3:**
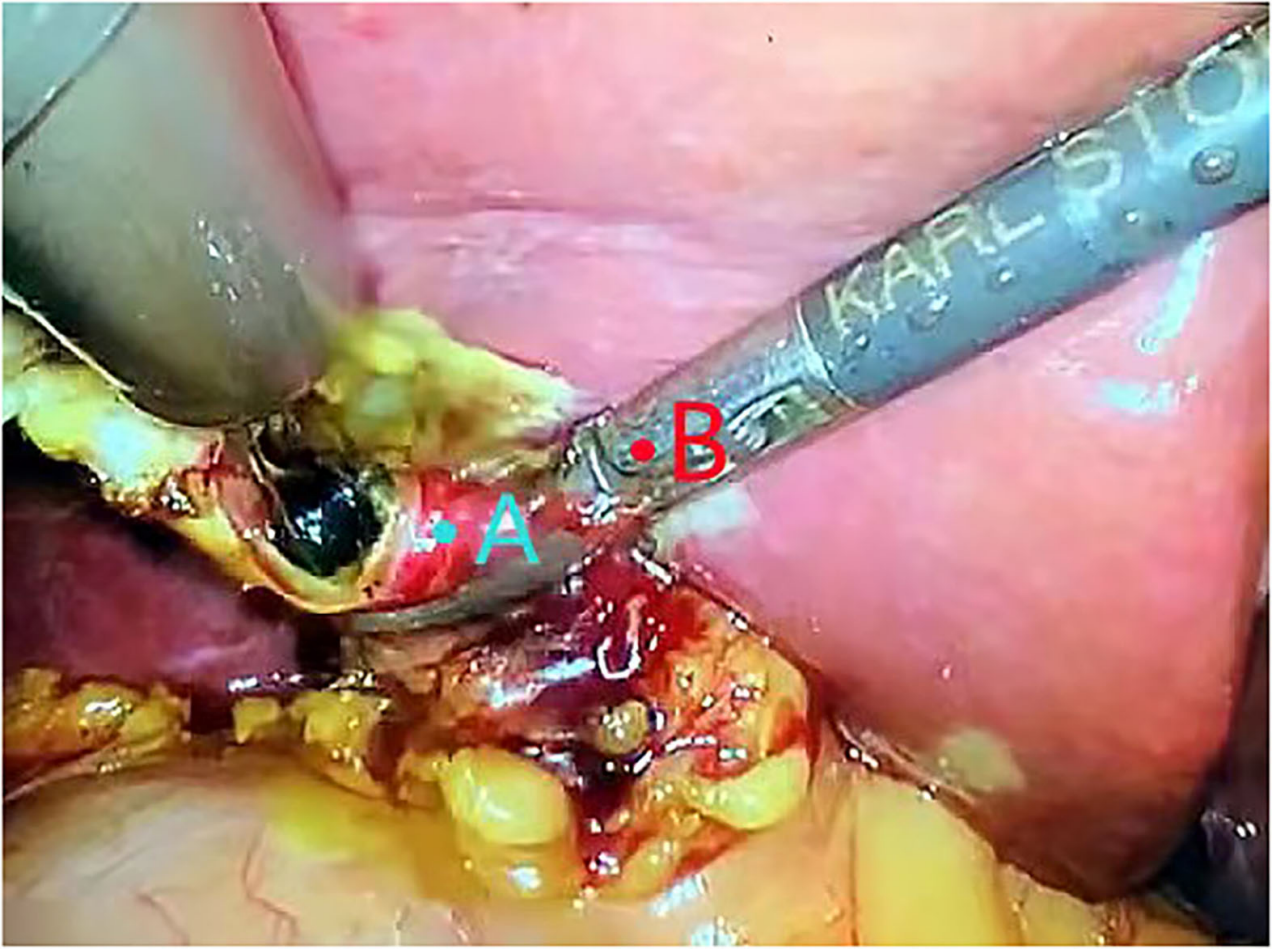
Using separation forceps to gently squeeze the stone outside the inlet to deform or break the stone and remove it. **(A)** cystic duct. **(B)** separation forceps.

If the choledocholithiasis diameter was smaller than the cystic duct diameter, the stone was removed using a stone basket. If the stone diameter was greater than the cystic duct diameter (<1.5 ×), the separation forceps was used to open and expand the common bile duct through the cystic duct incision; then, the stone basket was used to extract the stone from the part. If the stone still could not be removed after expansion, the separation forceps was used to gently squeeze the stone outside the inlet to deform or break the stone and remove it (Figure 3).

**Figure 4:**
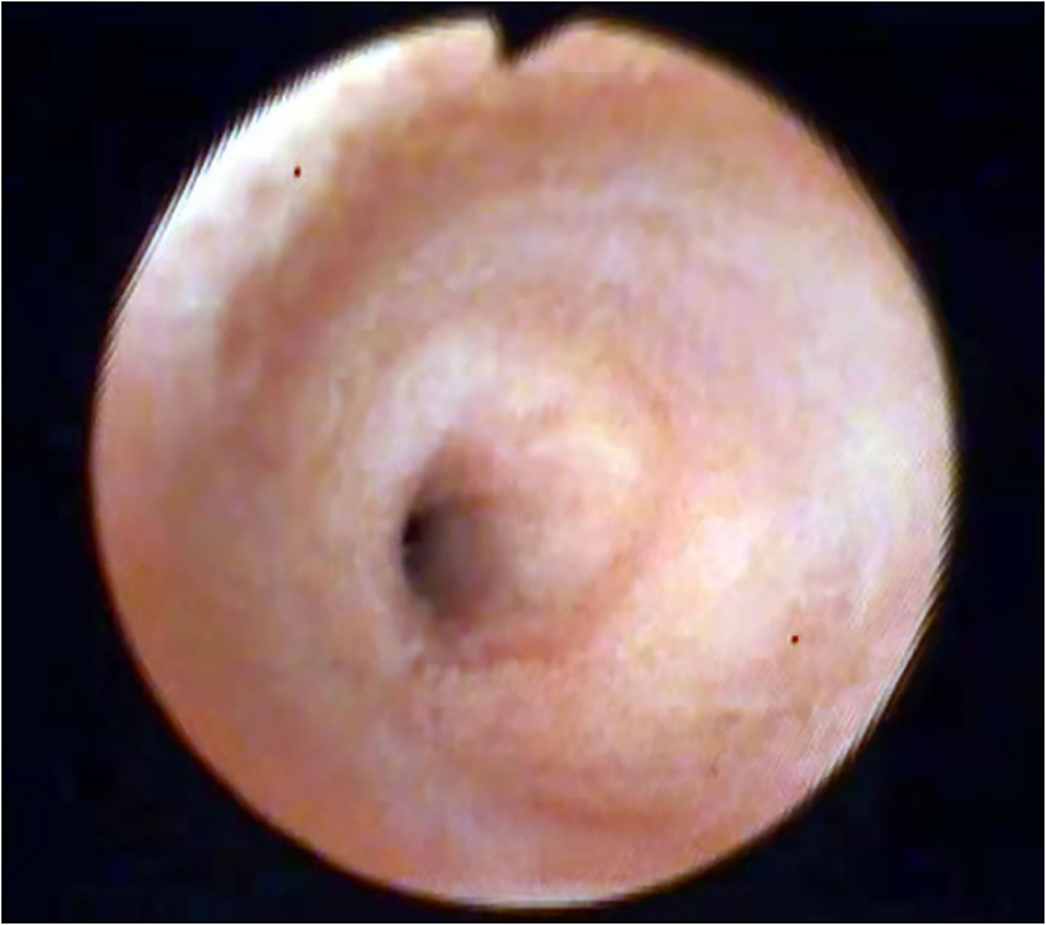
Observe the contraction of the duodenal sphincter.

Broken stones can be explored and removed using ultrathin choledochoscope several times. Then check whether there are residual stones at the lower end of the common bile duct, and observe the contraction of the duodenal sphincter (Figure 4).

After calculi removal, the choledochoscope was removed; the gall bladder was then removed retrogradely. Finally, the transverse section of the gall bladder duct was lifted, and two surgical clamps (Hemo-Lock) were placed between it and the common bile duct to close the gall bladder duct.

After rinsing the surgical area, the instrument suture puncture hole was pulled out. Generally, an abdominal drainage tube is not placed. In the case of severe cholecystitis symptoms, such as severe edema of the gallbladder and its duct as well as further blood oozing, are discovered during the operation, the minimally invasive surgery is not excessively pursued. Patient safety is the most important factor, and an abdominal drainage tube can be placed in this case. If there is no obvious exudation, bleeding, and biliary leakage, the tube can be removed within 1–2 days after operation. Patients without a drainage tube can be discharged after the second day of postoperative computed tomography examination with no abnormality. If patients experience abdominal discomfort (abdominal distension, abdominal pain, etc.), they can be discharged after 1–2 days of observation.

Variables used in the current analysis included demographic information, surgery duration, postoperative hospital stay, surgical outcome, and short-term and long-term complications. The patient postoperative biliary function was also evaluated according to the complications and postoperative results (6), and the surgical results were classified as (1) very good, (2) good, (3) average, and (4) poor. These results were used to evaluate biliary tract function and biliary tract function represented by Clavien–Dindo classification status, respectively (7).

The follow-up was performed at 1, 3, and 6 months after the operation, and then every 6 months. The purpose of the follow-up is to detect postoperative complications and assess the patient postoperative biliary function through early examination. Follow-up tests included abdominal ultrasound, computed tomography, magnetic resonance imaging, and blood biochemistry. The longest follow-up duration was 5 years.

## Statistical Analysis

SPSS (version 22.0) was used for data statistics. Continuous data were reported as mean ± standard deviation, and classified data as numbers with or without percentages. As all data were descriptive, the *p*-values were not reported.

## Results

Due to the strict inclusion and exclusion criteria, the number of cases included in the present study was low. All 47 enrolled patients (24 males and 23 females, with an average age of 48.3 years) successfully received Laparoscopic Transcystic Common Bile Duct Exploration (LTCBDE), without intraoperative changes or conversion to laparotomy. The operative success rate was 100%, the mean operation time was 65.5 ± 15.8 min, the mean postoperative hospital stay duration was 3.2 ± 2.1 days, and the overall stone clearance rate was 100% (Table 1).

**Table 1:** Patients information and perioperative situation (*n* = 47).

**Category**	**Data**
Age	48.3 ± 11.8
**Gender**	
Male	24
Female	23
Stone size (mm)	5.2 ± 3.8
Number of stones	2~6
The operation time (h)	65.5 ± 15.8
Postoperative hospital stay (d)	3.2 ± 2.1

*Data were expressed as mean ± standard deviation or numbers (percentage)*.

No patient developed postoperative bleeding, biliary leakage, or abdominal infection, and only 1 patient developed transient fever, which was considered mild cholangitis; the fever improved after symptomatic treatment. Two patients had severe chronic cholecystitis, intraoperative adhesions and bleeding on the wound surface, abdominal drainage tubes were placed for 2–3 days after operation to ensure patient safety.

No patient experienced postoperative bleeding, severe cholangitis, biliary pancreatitis, wound infection, or other complications. All patients participated in long-term follow-up with a mean duration of 32.5 ± 18.6 months (12–60 months). Biliary duct ultrasound and MRCP during the follow-up showed no bile duct stenosis or residual stones.

Overall, 38 patients (80.8%) had a very good outcome, 7 (14.9%) had a good outcome, 2 (4.3%) had an average outcome, and 0 (0%) had a poor outcome. The satisfaction rate (very good + good outcome) was 95.7% (Table 2).

**Table 2:** Follow-up information of patients (*n* = 47).

**Category**	**Data**
**Short-term complications**	
Transient fever	1 (2.1)
**Long-term complications**	
Cholangitis	0
Pancreatitis	0
Infection of incisional wound	0
Diarrhea	0
Recurrence of calculus	0
**Operation result**	
Very good	38 (80.8)
Good	7 (14.9)
Average	2 (4.3)
Poor	0 (0)

*Data were expressed as numbers (percentage)*.

## Discussion

Our recent study showed a success rate of 93.7 in the use of ultrathin choledochoscope and the holmium laser lithotripsy system for cholangiolithiasis treatment (6). However, since the holmium laser can no longer be used after adopting inclusion and exclusion criteria, the success of LTCBDE can only be achieved through the isolated use of ultrathin choledochoscope technology.

Several recent publications consider LTCBDE a safe and effective surgical procedure (8–10). The transcystic duct technique used in their study also included an incision along the cystic duct into the common bile duct [microincision, the cystic duct incision at the junction, with the common bile duct only extending 3–5 mm at the lateral edge of the common bile duct (CBD)], thus increasing the surgery success rate (9) (however, there is a risk of bile leakage) or microincision combined with laser lithotripsy for treatment (10).

In our exploration and lithotomy surgery, it is necessary to completely enter the common bile duct through the natural orifice of the cystic duct. The holmium laser is not used in the common bile duct; thus, there will be no incision, burning, or other invasive injuries to this area. There will also be no risk of bile leakage caused by re-suturing. This is all on account of ultrathin choledochoscope application. The use of the ultrathin choledochoscope can facilitate LTCBDE completion and avoid conversion to choledochotomy as well as associated complications.

The study conducted by Fang et al. (11) showed a stone clearance rate of 100% in 205 patients with gallstones and CBD stones treated using cystic duct microincision laser lithotripsy. The use of laser lithotriptomy could improve the stone clearance rate; meanwhile, mechanical lithotriptomy was used to remove stones with diameters larger than those of the cystic duct (see Patients and methods for detailed methods) in the present study. The stone clearance rate of the ultrathin choledochoscope LTCBDE (used alone) reached 100%.

Other studies have used t-incision of the cystic duct for LTCBDE. However, this method also requires incision suturing, carries the risk of bile leakage, and requires a longer abdominal drainage time. There is also the occurrence of postoperative mild –moderate pancreatitis (10). Kim et al. also provided a stone removal technique via cutting the lateral wall of the cystic duct and the common hepatic duct in a Calot triangle V shape. However, this method cannot guarantee the integrity of the common bile duct. Furthermore, laparoscopic suturing is difficult, and there are risks of bile leakage and common bile duct stenosis (12).

Two major LTCBDE difficulties (1) the placement of the choledochoscope into the cystic duct and (2) the passage through the cystic duct. Use of the ultrathin choledochoscope solves both difficulties (6).

The technical advantages of using single ultrathin choledochoscope are: (1) the surgery is minimally invasive; (2) cholecystectomy and common bile duct exploration are performed in one step; (3) Papilla function can be observed; (4) the common bile duct is not cut, and no continuous damage and injury is caused in this area. There is also no risk of common bile duct stenosis and bile duct leakage; (5) there is no risk of bile leakage from cystic duct incision without suture; (6) there is no need to apply the T-tube; (7) the abdominal drainage tubes are rarely placed; (8) a smaller postoperative incision can reduce patient pain; (9) the recovery is faster; (10) the hospital stay is shorter; (11) the medical costs are lower (13, 14); and (12) the patient quality of life is improved. Biliary tract function was maintained well during long-term follow-up in most patients.

Although scholars have shown that there are many methods for treating choledocholithiasis, no single method has been found to have obvious advantages compared with other methods so far (15). The present study emphasized the role of the inclusion and exclusion criteria, which is to study whether ultrathin choledochoscope technology can avoid difficulties and complications in the above-mentioned studies under controllable factors and conditions. A single-ultrathin-choledochoscope technique may be used to achieve a better surgical procedure by developing corresponding standards.

In conclusion, the use of ultrathin choledochoscope alone has a high application value. It is safe and effective in the treatment of cholecystolithiasis combined with choledocholithiasis through the use of LTCBDE. After strictly following the inclusion criteria and exclusion criteria, the stone extraction success rate can reach 100%.

## Data Availability Statement

The original contributions presented in the study are included in the article/supplementary material, further inquiries can be directed to the corresponding author.

## Ethics Statement

The study was conducted in accordance with the Declaration of Helsinki (as was revised in 2013). The study was approved by Ethics Committee of the First Medical Center, Chinese People's Liberation Army General Hospital.

## Author Contributions

YL and J-HL: conception and design of the research. YL and H-TX: critical revision of the manuscript for intellectual content. H-TX: writing of the manuscript. TY: statistical analysis. TY and XM: acquisition of data. YL: Analysis and interpretation of the data. All authors read and approved the final draft.

## Conflict of Interest

The authors declare that the research was conducted in the absence of any commercial or financial relationships that could be construed as a potential conflict of interest.

## Publisher's Note

All claims expressed in this article are solely those of the authors and do not necessarily represent those of their affiliated organizations, or those of the publisher, the editors and the reviewers. Any product that may be evaluated in this article, or claim that may be made by its manufacturer, is not guaranteed or endorsed by the publisher.
